# Unveiling rupture risk and clinical outcomes in midline aneurysms: A matched cohort analysis investigating the impact of localization within the anterior or posterior circulation

**DOI:** 10.1007/s10143-024-02310-6

**Published:** 2024-02-07

**Authors:** Vanessa M. Swiatek, Amir Amini, Celina E. Sandalcioglu Ortuño, Lena Spitz, Karl Hartmann, Ali Rashidi, Klaus-Peter Stein, Sylvia Saalfeld, I. Erol Sandalcioglu, Belal Neyazi

**Affiliations:** 1 Department of Neurosurgery, Otto-Von-Guericke University, Leipziger Str. 44, 39120 Magdeburg, Saxony-Anhalt Germany; 2 Department of Simulation and Graphics, Otto-Von-Guericke University, Universitätsplatz 2, 39106 Magdeburg, Saxony-Anhalt Germany; 3 Research Campus STIMULATE, Otto-Hahn-Str. 2, 39106 Magdeburg, Saxony-Anhalt Germany; 4 Department of Informatics and Automatisation, Technical University Ilmenau, Ehrenbergstr. 29, 98693 Ilmenau, Thuringia Germany

**Keywords:** Midline aneurysms, Anterior communicating artery aneurysm, Basilar tip aneurysm, Rupture risk, Semi-automatic neck reconstruction, Case-based reasoning

## Abstract

Intracranial aneurysms (IAs) located in the anterior and posterior circulations of the Circle of Willis present differential rupture risks. This study aimed to compare the rupture risk and clinical outcomes of anterior communicating artery aneurysms (AcomA) and basilar tip aneurysms (BAs); two IA types located along the midline within the Circle of Willis. We retrospectively collected data from 1026 patients presenting with saccular IAs. Only AcomA and BAs with a 3D angiography were included. Out of 186 included IAs, a cohort of 32 BAs was matched with AcomA based on the patients’ pre-existing conditions and morphological parameters of IAs. Clinical outcomes, including rupture risk, hydrocephalus development, vasospasm incidence, and patients’ outcome, were compared. The analysis revealed no significant difference in rupture risk, development of hydrocephalus, need for ventricular drainage, or vasospasm incidence between the matched AcomA and BA cohorts. Furthermore, the clinical outcomes post-rupture did not significantly differ between the two groups, except for a higher Fisher Grade associated with BAs. Once accounting for morphological and patient factors, the rupture risk between AcomA and BAs is comparable. These findings underscore the importance of tailored management strategies for specific IA types and suggest that further investigations should focus on the role of individual patient and aneurysm characteristics in IA rupture risk and clinical outcomes.

## Introduction

Understanding the differences in rupture risk between intracranial aneurysms (IA) located in the anterior and posterior circulations of the Circle of Willis is essential for the clinical management of IA patients. The localization of an IA influences its likelihood to rupture [3, 5, 6, 11, 12, 19–24, 27–30, 32]. Previously published studies suggest that posterior circulation IAs present with a higher rupture risk than anterior circulation IAs [8, 28, 31, 32]. The suggested increased rupture risk of basilar tip aneurysms (BA) might be due to factors related to their specific localization within the posterior circulation. These factors include distinctive hemodynamic stress, vessel wall characteristics, and micro-environmental factors. Furthermore, the posterior circulation intricate arrangement and proximity to critical neurological structures worsen the consequences of a rupture event [5, 23, 28, 31]. However, anterior communicating artery aneurysms (AcomA), while exhibiting a lower rupture risk than posterior circulation IAs, still hold clinical importance due to their high prevalence and their own elevated rupture risk relative to other anterior circulation IAs. A rupture close to vital cerebral structures of the anterior and middle cranial fossa can likewise lead to significant clinical complications [2, 4, 11, 24, 27, 33].

Given their midline localization in the Circle of Willis, BAs and AcomA serve as an essential subgroup of IAs. The distinct positioning of AcomA and BAs along the midline within the Circle of Willis creates an opportunity to study if the localization within the anterior or posterior circulation represents an independent risk factor for rupture, rupture severity, and subsequent clinical complications.

This investigation presents a detailed comparative analysis of AcomA and BAs, using a matched cohort with similar clinical and morphological characteristics. To ensure the robustness of the cohort matching, we incorporated a comprehensive set of 15 clinical indices and 21 morphological variables, all acquired retrospectively. The main aim of this research was to determine if there is a difference in the risk of rupture, subsequent complications, and clinical outcome scores between IAs in the anterior and posterior circulation within a matched cohort of AcomA and BA.

## Materials and methods

For this study, a retrospectively collected database containing a total of 1026 patients with 1469 saccular IAs who presented at the Department of Neurosurgery of the Otto-von-Guericke University Hospital, Magdeburg, Germany, between 2000 and 2018 due to this diagnosis was initially screened for the following inclusion criteria:

1. Availability of 3D angiography with the possibility of semi-automatic reconstruction of the morphological characteristics of the IA
2. Localization of the aneurysm at the anterior communicating artery (Acom) or the basilar tip

**Fig. 1:**
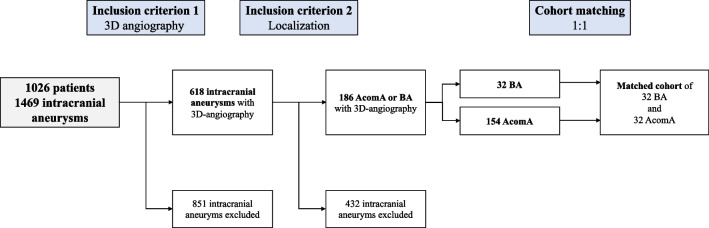
Flow chart depicting the determination of the study cohort according to the inclusion criteria (AcomA, anterior communicating artery aneurysm; BA, basilar tip aneurysm)

The ethics committee of the Otto-von-Guericke University waivered the analysis of retrospectively collected data. All the procedures being performed were part of the routine care. After defining the cohort using the inclusion criteria described above, an AcomA matched with respect to the IA morphology and pre-existing medical records of the patient was selected for each included BAs (Fig. 1).

### Data acquisition

The clinical data for this investigation were extracted retrospectively through an in-depth analysis of patient medical records, anamneses, medication registries, and existing diagnostic imaging. Evaluation of patient medical history included an assessment of prevalent cardiovascular diseases, associated risk factors, and other critical health conditions, such as malignant neoplasms or significant autoimmune disorders necessitating immunosuppressive therapy. Pertaining to the natural progression and management of IAs, extensive data were gathered from the results of diagnostic imaging, aneurysm-specific risk factors, and evaluations of patient clinical outcomes post-aneurysm treatment or rupture. The parameters obtained are enumerated in Table 1 and have been defined as follows:

**Table 1:** List of examined clinical parameters and their definitions [1, 9, 10, 12–16, 26]

Parameter category	Parameters/definitions
Epidemiological data	Age (defined as age at diagnosis)
	Gender (defined as biological gender)
Medical history	Hypertension (defined as documented diagnosis or intake of antihypertensive medication)
	Diabetes mellitus (defined as a documented diagnosis of type 1 or 2 diabetes or intake of oral antidiabetics or insulin)
	Hyperlipidemia (defined as documented diagnosis or intake of medication lowering the lipid or cholesterol levels in the blood)
	Peripheral arterial disease (defined as documented diagnosis or imaging finding)
	Heart disease (defined as a documented diagnosis of myocardial infarction, coronary artery disease, cardiac arrhythmia, or other heart diseases)
	Ischemic stroke (defined as documented diagnosis or imaging finding at admission)
	Thrombosis (defined as documented diagnosis)
	Malignant tumor disease (defined as documented diagnosis regardless of affected organ)
	Autoimmune disease (defined as documented diagnosis with need of immunosuppressive therapy)
	Obesity (defined as a documented body mass index of > 30 kg/m^2^)
	Nicotine abuse (defined as ex-nicotine abuse or continued nicotine abuse)
	Alcohol abuse (defined as consumption of > 50 g of alcohol per week)
	Contraceptive use and intake of hormone replacement products (at time of diagnosis, extracted from medical records or medication plans)
CT-imaging parameters	Type of bleeding, shifting of the midline, intraventricular hemorrhage, hydrocephalus, and ischemia (in the first CT-scan after SAH)
Aneurysm-related parameters	Rupture status (defined by assessment of intraoperative findings, imaging findings, and bleeding patterns in CT)
	Multiplicity (defined as > / = 2 intracranial aneurysms)
	Aneurysm localization (defined by assessment of angiography)
Clinical scores	Glasgow coma scale at admission and discharge (defined by neurological examination at admission and discharge)
	The modified Rankin scale at discharge (defined by neurological examination at discharge)
	The Hunt and Hess grade, WFNS score, and Fisher Grade at admission (defined by neurological examination at admission and imaging findings)
Treatment-related parameters	Previous and current treatment and treatment-modality (extracted from medical records)
Complication-related parameters	Hydrocephalus, placement of an external ventricular drainage, or ventriculoperitoneal shunt (extracted from medical records)
	Vasospasm, method of Vasospasm-detection, and treatment via endovascular spasmolysis (extracted from medical records)
Follow-up data	Time of follow-up, perfusion of the aneurysm, and the modified Rankin scale at follow-up (extracted from medical records)

In the framework of this study, rigorous definitions for hydrocephalus, vasospasm, and delayed cerebral ischemia are essential. Hydrocephalus was delineated based on serial CT imaging of the neurocranium, specifically identifying an expansion of the ventricular system. Vasospasm was ascertained through two methodologies: firstly, by the detection of increased cerebral blood flow velocities via daily transcranial Doppler examinations, and secondly, through direct visualization of vasospasms in digital subtraction angiography (DSA). Delayed cerebral ischemia was characterized as a territorial cerebral infarction attributable to vasospasms, distinctly excluding infarcts arising as sequela of postoperative or postinterventional interventions.

### Morphological analysis

**Fig. 2:**
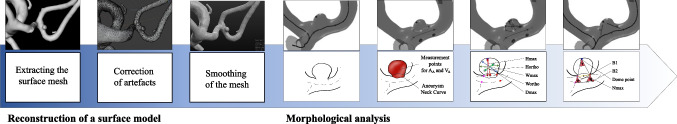
Depiction of the workflow for segmenting a three-dimensional vascular model from 3D angiography datasets, along with an illustration of the ensuing morphological analysis facilitated by a semi-automatic neck reconstruction and definition of the key morphological parameters

Based on the restructured and digitally subtracted 3D rotational angiography dataset, we derived 3D surface models following the methodology proposed by Saalfeld and colleagues (Fig. 2) [21]. Subsequently, a semi-automatic segmentation of the neck curve was implemented, enabling the automated derivation of 21 morphological parameters (Table 2, Fig. 2) [22]. Unlike manual measurement methods, this approach enhances objective scrutiny of the 3D vessel and offers the capability to analyze extensive data volumes swiftly and efficiently.

**Table 2:** Definition of the 21 semi-automatically extracted morphological parameters [7, 17, 18, 22, 30]

Morphological parameters	Definition
Hmax	Maximum height of the aneurysm
Wmax	Maximum width of the aneurysm perpendicular to Hmax
Dmax	Maximum diameter of the aneurysm
Hortho	Height of the aneurysm; measured vertically to the aneurysm neck
Wortho	Maximum width of the aneurysm perpendicular to Hortho
Nmax	Maximum diameter of the aneurysm neck
Navg	Average diameter of the aneurysm neck
AR 1	Aspect ratio 1 (Hortho / Nmax)
AR 2	Aspect ratio 2 (Hortho / Navg)
EI	Ellipticity Index (1–18^(1/3) V_CH^(2/3) / A_CH)
NSI	Non-sphericity Index (1–18^(1/3) V^(2/3) / A_A_)
UI	Undulation Index (1-V / V_CH)
*A*_A_	Surface of the aneurysm
Ostium Area 1	Surface of the aneurysm ostium
Ostium Area 2	Surface of the aneurysm ostium; the neck curve projected onto a plane
*V*_A_	Volume of the aneurysm
V_CH	Volume of the convex hull of the aneurysm
A_CH	Surface of the convex hull of the aneurysm
Alpha	Angle at point B1 describing the angle from the baseline to the dome point
Beta	Angle at point B2 describing the angle from the baseline to the dome point
Gamma	Angle at the aneurysm dome

The extraction and definitions of the here mentioned parameters are illustrated in Fig. 2

### Cohort matching

**Fig. 3:**
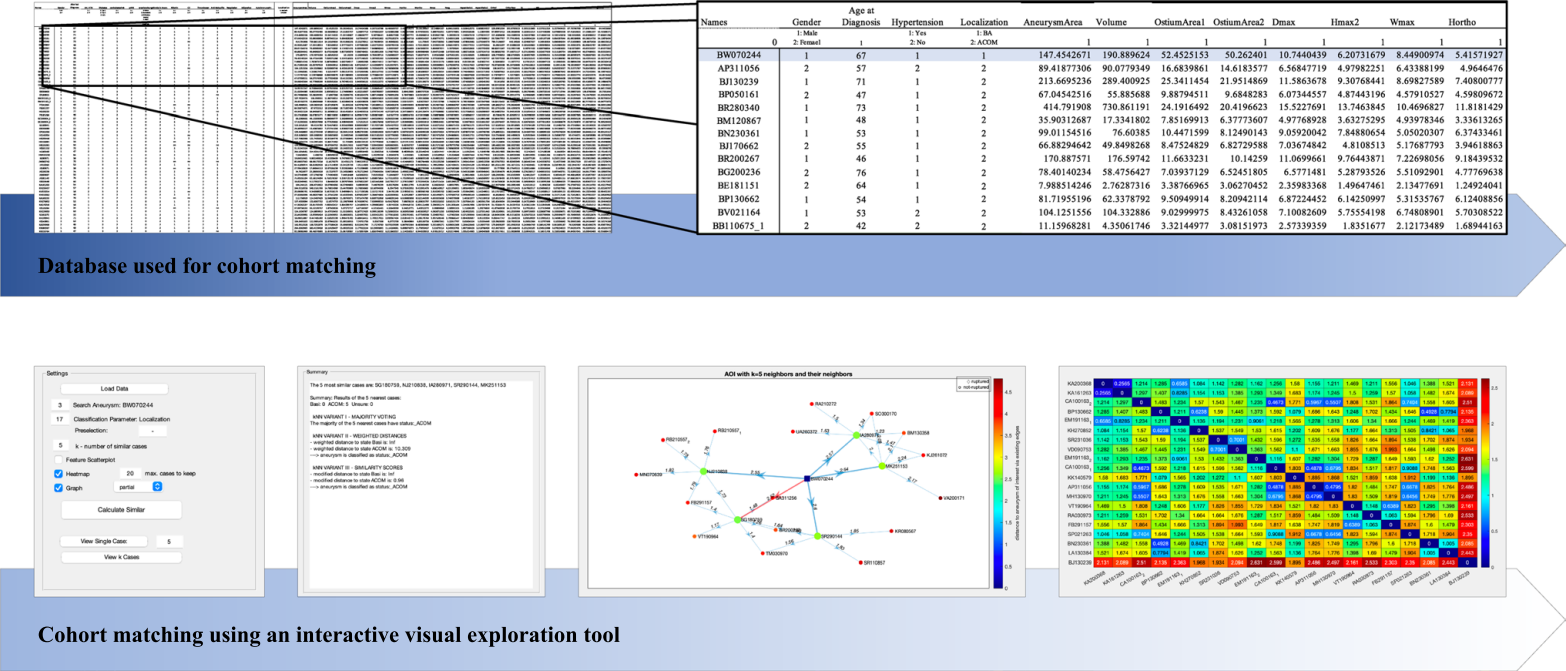
The upper part of the figure illustrates the utilized database, which includes 15 clinical and 21 morphological parameters for matching and serves as the basis for cohort matching using an Interactive Visual Exploration Tool. The excerpt from the database highlights the AOI in blue, with a selection of 154 AcomA displayed below. From this subset, the five most similar IAs are subsequently chosen. The second part of the illustration presents the settings panel, where the Excel file is loaded, and the AOI, the variable for matching, and the number of cases are selected. The Interactive Visual Exploration Tool first provides a text panel displaying the names of the five most similar IAs and the three mentioned k-NN classifiers. The results are then visualized using a directed graph panel and an interactive heat map [25]

In the present study, 32 BAs were matched with AcomA, considering the patients’ pre-existing conditions and the 21 morphological parameters (Table 2) that were extracted. This methodical pairing aimed to attenuate the influence of these significant risk factors on the investigated outcome variables, thereby permitting a more equitable comparison between midline aneurysms situated in the anterior and posterior circulation of the Circle of Willis. Due to the high number of parameters intended for matching, an interactive visual exploration tool designed for case-based reasoning of IAs introduced by Spitz et al. was used [25]. First, we filled in a pre-existing Excel file with the aforementioned parameters for all 154 AcomA and a specific BA. This Excel file was uploaded into the interactive visual exploration tool (Fig. 3). The selected BA, defined as the “aneurysm of interest” (AOI), was replaced with the next BA after each successful matching, and the matching process was initiated again.

For IA matching, case-based reasoning was employed as the methodology to define the most similar cases to the selected BA, measured by the k-nearest neighbor-based (k-NN) classification. For the k-NN classification, a selected BA (AOI) was compared to a reference database containing the AcomA enrolled in this study. To predict the most similar AcomA to the BA, the approach considers the *k* (here defined as five) most similar cases. The values of each feature are normalized using standardization, specifically the *Z*-score standardization. The dissimilarity between two IAs, *x* and *q*, is calculated using the following formula:

$$\mathrm{dist}(x,q)=\surd\left(\sum\mathrm l=1N\left(\mathrm{fwl}\ast{\left(\mathrm{xl}-\mathrm{ql}\right)^\wedge2}\right)\right)$$

In this equation, *N* represents the number of features, xl denotes the value of the l-th feature of aneurysm *x*, and fwl represents the weight assigned to the feature. By default, all feature weights are set to 1.

The first variant of the classifier used in this study is a simple k-NN classifier. The algorithm calculates the dissimilarity between the AOI and all IAs in the database. Then, it selects the *k*, here set to five, nearest IAs and determines the nearest neighbors. In the ordinary k-NN classifier, every nearest neighbor has an equal impact on the classification, regardless of its actual distance to the AOI. To address this, the second variant incorporates the actual distances as weights by assigning a weight to each near IA which is inversely proportional to the distance. The third variant of the k-NN-based classifier follows a similar approach but with the additional step of normalizing all distances using min–max scaling into the range of [0,1].

In addition, to facilitate interactive exploration and analysis of the data, a visual analytics framework was developed. This framework integrated various visualization techniques, such as a summary panel, a directed graph panel, and an interactive heat map. These visualizations allow users to examine and compare the features of different IAs and to identify the most similar comparative AcomA for selected BAs (Fig. 3).

**Fig. 4:**
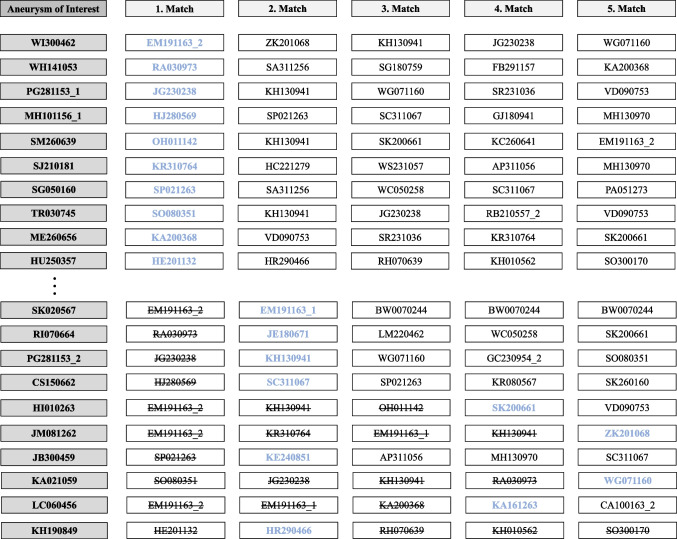
Illustration of the matching process highlighting the procedure performed in cases where an already matched AcomA was selected as the most similar IA for more than one AOI. Initially, a total of 10 AcomA were identified as the nearest aneurysms, and they were already included in the cohort through matching with another AOI, as depicted in the cases WI300462-HU250357. To ensure an accurate selection process, subsequent matches were considered in descending order (cases SK020567-KH19084). The goal was to find the best match that had not been assigned to another AOI. In this manner, the most suitable AcomA aneurysms were chosen for inclusion in the cohort. Among the cases analyzed, the second-nearest AcomA was selected in 6 instances. In 2 cases, the fourth-nearest AcomA was chosen, while in 2 other cases, the fifth-nearest AcomA was selected

During the matching process, an AcomA was identified as the nearest IA in 10 cases, which had already been included in the cohort through matching with another AOI. In these cases, the subsequent matches were considered in descending order, and the best match that had not been assigned to another IA was selected. Therefore, in 6 cases, the second-nearest AcomA was chosen, in two cases the fourth-nearest, and in two cases the fifth-nearest AcomA was selected and included in the matched cohort of AcomA (Fig. 4). As a result, a total of 32 distinct AcomA were statistically compared with the 32 BAs at the end.

### Statistical analysis

Statistical analyses were conducted utilizing IBM SPSS Statistics 29. For numerical variables, chi-square tests were deployed, or Fisher’s exact test was employed in cases where the expected cell frequency in one or more cells was less than 5. In the case of ordinal or metric variables, the Kolmogorov–Smirnov test was initially applied to assess the normality of distribution, followed by Levene’s test to evaluate the homogeneity of variances.

Subsequently, data that did not follow a normal distribution were analyzed with the Mann–Whitney *U* test. For data exhibiting a normal distribution, either a *t*-test or Welch’s test was performed, as appropriate.

## Results

### Cohort overview

In the comprehensive cohort encompassing 186 IAs, a comparative analysis between 154 AcomA and 32 BAs was conducted. This analysis unveiled distinctive patterns in terms of gender distribution, prevalence of autoimmune diseases, usage of hormonal medications, proportions of ruptured IAs, and the incidence of multiple IAs. It was discerned that autoimmune diseases were more prevalent in patients presenting with AcomA, whereas hormonal medication intake was found to be more common in patients with BAs. Moreover, a higher frequency of ruptured IAs was associated with localization at the Acom, whereas BAs exhibited a higher tendency for multiplicity (Table 3). Upon the implementation of cohort matching, the analysis demonstrated no discernible differences between the two groups in relation to the parameters under investigation (Table 3).

**Table 3:** Comparison of epidemiological data and pre-existing conditions, rupture status, and aneurysm multiplicity across the entire cohort and the matched sub-cohort, including tests for significant differences between cohorts

	Whole cohort (*n* = 186)			Matched cohort (*n* = 64)		
	AcomA (*n* = 154)	BA (*n* = 32)	Statistical analysis	AcomA (*n* = 32)	BA (*n* = 32)	Statistical analysis
Gender	M = 69; F = 85	M = 7; F = 25	*p* = 0.008*	M = 7; F = 25	M = 7; F = 25	*p* = 1*
Age	53.9 years	56.8 years	*p* = 0.149**	55.2 years	56.8 years	*p* = 0.248**
Hypertension	108 (70.1%)	23 (71.9%)	*p* = 0.724*	25 (78%)	23 (71.9%)	*p* = 0.714*
Diabetes mellitus	13 (8.4%)	4 (12.4%)	*p* = 0.503***	2 (6.2%)	4 (12.4%)	*p* = 0.485***
Hyperlipidemia	21 (13.6%)	7 (21.9%)	*p* = 0.083***	6 (18.8%)	7 (21.9%)	*p* = 0.873***
Peripheral arterial disease	3 (2%)	2 (6.2%)	*p* = 0.187***	0 (0%)	2 (6.2%)	*p* = 0.238***
Heart disease	24 (15.6%)	4 (12.4%)	*p* = 0.531***	2 (6.2%)	4 (12.4%)	*p* = 0.361***
Ischaemic stroke	9 (5.8%)	1 (3.1%)	*p* = 0.489***	0 (0%)	1 (3.1%)	*p* = 0.492***
Thrombosis	6 (4%)	0 (0%)	*p* = 0.350***	1 (3.1%)	0 (0%)	*p* = 0.738***
Malignant tumor disease	5 (3.3%)	3 (9.4%)	*p* = 0.711***	1 (3.1%)	3 (9.4%)	*p* = 0.488***
Autoimmune disease	17 (11%)	0 (0%)	*p* = 0.042***	9 (28.1%)	0 (0%)	*p* = 0.508***
Obesity	50 (32.5%)	12 (37.5%)	*p* = 0.762*	1 (3.1%)	12 (37.5%)	*p* = 0.59*
Nicotine abuse	79 (51.3%)	16 (50%)	*p* = 0.689*	18 (56.3%)	16 (50%)	*p* = 0.748*
Alcohol abuse	21 (13.6%)	5 (15.6%)	*p* = 0.517*	1 (3.1%)	5 (15.6%)	*p* = 0.142***
Hormonal medication	0 (0%)	2 (6.3%)	*p* = 0.027***	0 (0%)	2 (6.3%)	*p* = 0.229***
Ruptured aneurysms	114 (74%)	15 (46.9%)	*p* = 0.005*	21 (65.6%)	15 (46.9%)	*p* = 0.206*
Multiple aneurysms	44 (28.6%)	16 (50%)	*p* = 0.012*	9 (28.1%)	16 (50%)	*p* = 0.127*
Aneurysm size (mean in cm)	5.7	5.7	0.928**	5.5	5.7	*p* = 0.397****

Significant results are highlighted in bold

*AcomA* anterior communicating artery aneurysm, *BA* basilar tip aneurysm

^*^chi-square test, **Mann–Whitney *U* test, ***Fisher exact test, ****t-test

### Outcome analysis

Subsequent statistical examination was bifurcated into two integral facets: firstly, we investigated the natural course of AcomA and BAs with a particular focus on rupture risk and the subsequent risk of complications, especially the development of hydrocephalus or vasospasm. Secondly, we endeavored to contrast the clinical presentations and outcomes post-rupture of these IAs.

**Fig. 5:**
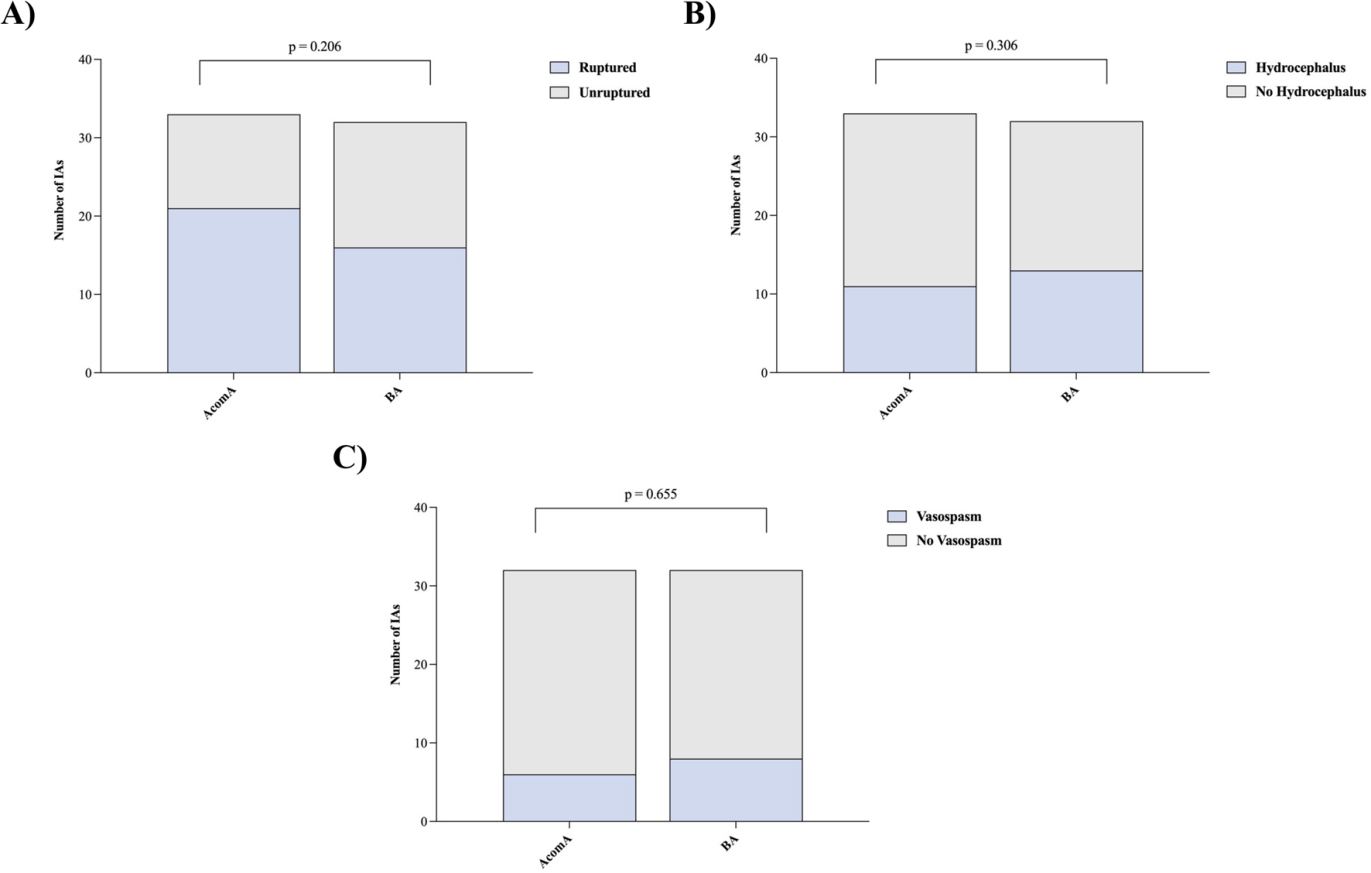
Illustration of **A** analysis of the rupture rate, **B** the incidence of hydrocephalus, and **C** vasospasm for the matched AcomA and BA cohorts (AcomA, anterior communicating artery aneurysm; BA, basilar tip aneurysm)

When comparing the matched cohorts of AcomA and BAs, no significant disparity was observed in terms of rupture risk (*p* = 0.206). Hydrocephalus defined as enlargement of the ventricular system on CT scan was diagnosed in 11 patients with AcomA and in 14 patients with BAs. External ventricular drainage was placed in 9 of the 32 patients with AcomA and in 11 of the 32 patients with BAs. Among these, a ventriculoperitoneal shunt was subsequently implanted in 4 patients from each cohort. Likewise, the development of hydrocephalus (*p* = 0.306), the necessity for external ventricular drainage placement (*p* = 0.457), or ventriculoperitoneal shunt insertion (*p* = 0.607) did not display any significant differences between the two cohorts (Fig. 5). Within the AcomA cohort, vasospasm was diagnosed in six patients—identification in one case was through transcranial doppler examinations, while the remaining five were confirmed via angiographic methods. Of these, two patients experienced delayed cerebral ischemia. In contrast, in the BA cohort, eight patients were diagnosed with vasospasm, with three detections via transcranial doppler examinations and five through angiography. Similarly, two patients in this group developed delayed cerebral ischemia. The incidence of vasospasm (*p* = 0.655), subsequent delayed cerebral ischemia (*p* = 0.467), or the requirement for spasmolysis (*p* = 0.156) were similarly indistinguishable between the two IA groups (Fig. 5). All patients diagnosed with vasospasm received prophylaxis with nimodipine, adhering to our institution’s protocol, both prior to the onset of vasospasms and during the vasospasm phase. Each patient underwent intensive conservative spasm therapy, particularly focusing on the consistent elevation of mean arterial pressure. In the AcomA cohort, one patient additionally underwent intra-arterial spasmolysis. Conversely, in the BA cohort, intra-arterial spasmolysis was performed on four patients.

**Fig. 6:**
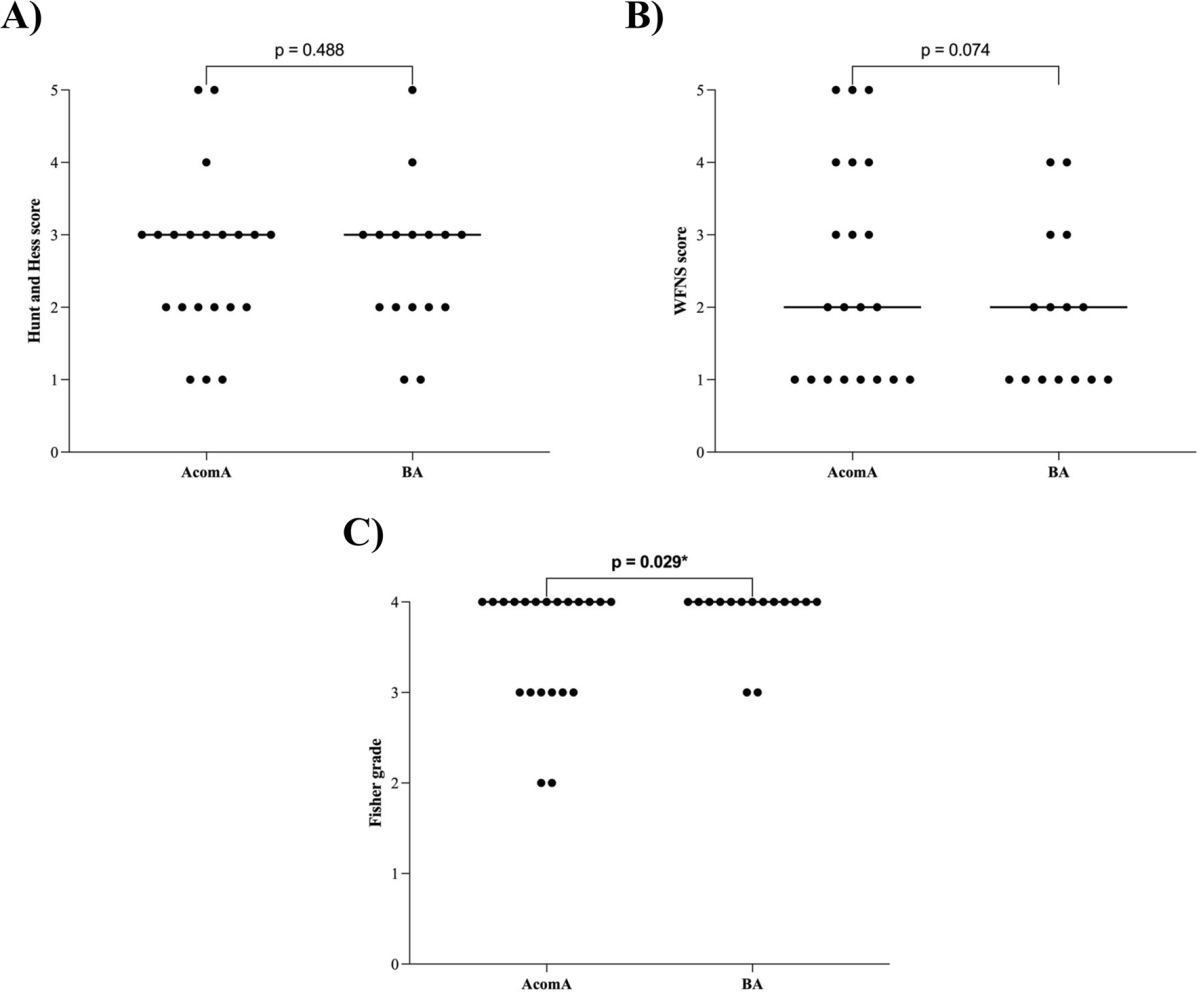
Illustration of **A** analysis of the Hunt and Hess score, **B** the WFNS score, and **C** the Fisher Grade for the matched AcomA and BA cohorts. Each point represents an analyzed case. The transverse bar, connecting several points of the same value, denotes the median value of the cohort (AcomA, anterior communicating artery aneurysm; BA, basilar tip aneurysm)

**Fig. 7:**
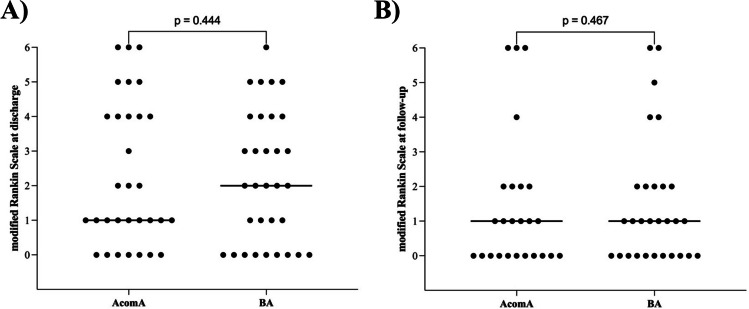
Illustration of analysis of the modified Rankin Scale (**A**) at discharge and **B** follow-up for the matched AcomA and BA cohorts. Each point represents an analyzed case. The transverse bar, connecting several points of the same value, denotes the median value of the cohort (AcomA, anterior communicating artery aneurysm; BA, basilar tip aneurysm)

In the subset of ruptured IAs, no significant disparities were noted in the clinical subarachnoid hemorrhage (SAH) scores, specifically, the Hunt and Hess score (*p* = 0.488) and the World Federation of Neurosurgical Societies (WFNS) score (*p* = 0.074) (Fig. 6). However, a statistically significant difference was observed in the Fisher Grade (*p* = 0.029), wherein BAs presented with a higher Fisher Grade upon initial CT scan examination (Fig. 6). For the entire set of IAs under study, no significant differences were discerned in the Glasgow Coma Scale (GCS) at both admission (*p* = 0.246) and discharge (*p* = 0.187), as well as in the modified Rankin Scale (mRS) at discharge (*p* = 0.444) and during follow-up evaluations (*p* = 0.467) (Fig. 7). The average follow-up duration for the AcomA cohort was 33 months, compared to 39 months for the BA cohort.

## Discussion

The aim of this study was to compare the rupture risks and clinical outcomes between patients with AcomA and BAs. Previous studies suggest that posterior circulation IAs, such as those found at the basilar tip, present a higher likelihood of rupture compared to anterior circulation IAs, such as those at the AcomA [8, 28, 31, 32]. This distinction in rupture risk is believed to be influenced by several factors specific to the posterior circulation.

One key factor contributing to the higher rupture risk of posterior circulation IAs might be a distinct hemodynamic stresses experienced in this region. The intricate arrangement of vessels and the complex flow patterns in the posterior circulation as well as distinct vessel wall characteristics subject IAs to unique mechanical forces that may predispose them to rupture. Furthermore, the posterior circulation is in close proximity to critical neurological structures, increasing the potential for severe consequence and leading to more severe clinical outcomes in the event of a rupture [5, 23, 28, 31].

While posterior circulation IAs exhibit a higher rupture risk compared to anterior circulation IAs, it is important to acknowledge the clinical significance of AcomA. AcomA carry significant clinical importance due to their high occurrence accounting for 15–37% of all IAs and their elevated rupture risk relative to other anterior circulation IAs [2, 4, 11, 24, 27, 33].

The unique midline localization of AcomA and BAs within the Circle of Willis presents an opportunity to investigate the potential influence of IA location in the anterior or posterior circulation as a significant factor. Understanding the impact of IA location in these distinct cerebrovascular territories is crucial for advancing the rupture risk assessment of IA patients.

In this study, a matched cohort approach was employed to ensure comparable clinical and morphological characteristics between AcomA and BA cases. Interestingly, following a careful cohort-matching process to mitigate the influence of confounding variables, no significant differences were observed in terms of rupture risk. This suggests that, once accounting for morphological and patient factors, the rupture risk between AcomA and BAs might be comparable and not directly influenced by IA localization in the anterior or posterior circulation. In the subset of ruptured IAs, no significant differences were found in the clinical subarachnoid hemorrhage scores, as indicated by the Hunt and Hess score and the World Federation of Neurosurgical Societies score. However, BAs exhibited a higher Fisher Grade upon initial CT scan examination, indicating a more severe subarachnoid hemorrhage [10, 12]. Interestingly, the incidence of vasospasm, delayed cerebral ischemia, or requirement for spasmolysis was not higher in BAs. Also, no difference in the development of hydrocephalus, need for external ventricular drainage, or ventriculoperitoneal shunt was observed. There were no significant differences in the Glasgow Coma Scale and modified Rankin Scale at admission, discharge, and follow-up evaluations.

For an accurate interpretation of these results, the following limitations need to be considered: Firstly, the retrospective design of our study limits our ability to fully understand the natural progression of untreated aneurysms, particularly in cases where unruptured aneurysms were treated. Secondly, the rarity of BAs led to a smaller cohort size in our study. This limitation could affect how broadly our findings apply to a wider population. Lastly, we employed a 1:1 matching approach to minimize variability. While this was beneficial for controlling certain aspects, it may not reflect the full diversity of clinical presentations in intracranial aneurysms.

However, our findings emphasize the importance of individualizing risk stratification and treatment strategies based on aneurysm location. Although the rupture risks were comparable between AcomA and BAs in this matched cohort study, the challenges in diagnosing and managing posterior circulation IAs, as well as the potential severity of rupture indicated by the higher Fisher Grade, highlight the need for careful clinical consideration. Future research should aim to further refine our understanding of the complex interplay of factors influencing rupture risk and outcomes in different cerebrovascular territories.

In conclusion, this comprehensive study provides valuable insights into the rupture risks and clinical outcomes of AcomA and BAs. The findings contribute to our understanding of the differences between anterior and posterior circulation IAs and underscore the importance of tailored management strategies for these specific IA types.

## Data Availability

The data supporting the findings of this study are not openly available due to reasons of sensitivity and are available from the corresponding author upon reasonable request.
